# CPAP indication based on clinical data and oximetry for patients with suspicion of obstructive sleep apnea: A multicenter trial

**DOI:** 10.5935/1984-0063.20190089

**Published:** 2019

**Authors:** Carlos Alberto Nigro, Eduardo Enrique Borsini, Eduardo Dibur, Luis Dario Larrateguy, Alexis Cazaux, Carlos Elias, Marcelino de-la-Vega, Cecilia Berrozpe, Silvana Maggi, Sofía Grandval, Hugo Cambursano, Daniela Visentini, Juan Criniti, Facundo Nogueira

**Affiliations:** 1 Hospital Alemán, Sleep Lab, Pneumonology - Buenos Aires - Buenos Aires - Argentina.; 2 Hospital Británico, Sleep Lab, Pneumonology - Buenos Aires - Buenos Aires - Argentina.; 3 Centro Privado de Medicina Respiratoria, Sleep Lab, Pneumonology - Paraná - Entre Rios - Argentina.; 4 Centro Dr. Lázaro Langer, Sleep Lab, Pneumonology - Córdoba - Córdoba - Argentina.; 5 Instituto Médico Insares, Sleep Lab, Pneumonology - Mendoza - Mendoza - Argentina.; 6 Hospital Privado Santa Clara de Asis, Sleep Lab, Pneumonology - Salta - Salta - Argentina.; 7 FLENI, Sleep Lab, Neurology - Buenos Aires - Buenos Aires - Argentina.; 8 CEMIC, Sleep Lab, Neurology - Buenos Aires - Buenos Aires - Argentina.; 9 Sanatorio San Lucas, Sleep Lab, Pneumonology - San Isidro - Buenos Aires - Argentina.; 10 Hospital Cetrángolo, Sleep lab, Pneumonology - Florida - Buenos Aires - Argentina.

**Keywords:** Continuous Positive Airway Pressure, Diagnosis, Oximetry, Obstructive Sleep Apnea, Ambulatory Monitoring

## Abstract

**Background and Objective:**

The usefulness of pulse oximetry for the management of obstructive sleep apnea is controversial. The aim of this study was to assess the accuracy for indication of Continuous Positive Airway Pressure (CPAP) treatment in patients with suspected obstructive sleep apnea (OSA) based on clinical and oximetry data as compared to polysomnography (PSG).

**Methods:**

This multicenter observational study involved seven sleep laboratories. Patients with suspicion of OSA who completed a standardized sleep questionnaire and a diagnostic PSG were enrolled. Eight observers logged on to a website independently and blindly. Seven observers only accessed the clinical data, curve and pulse oximetry results (Os-SO2-test method), while the eighth observer had full access to all indicators of PSG (O-PSG-reference method). Once observers assessed the information available on the website, they had to choose between three CPAP treatment options (yes/no/do not know) based on their knowledge and criteria.

**Results:**

411 subjects (228 men), median age 54 years, were available for evaluation. Os-SO2 had lower sensitivity (S), greater specificity (Sp) and positive likelihood ratio (PLR) to prescribe CPAP in patients more symptomatic (Epworth Sleepiness Scale-ESS > 10 or comorbidities) than those with fewer symptoms (ESS < 11 without comorbidities) (S 45-75% versus 45-91%, *p* 0.028); Sp 93.8-100% versus 68.5-96.6%, *p* 0.004; PLR > 10 versus 2.9-17, *p*<0.01).

**Conclusions:**

Due to its low false positive rate, a strategy based on pulse oximetry and clinical data was a consistent tool to indicate CPAP treatment in most symptomatic patients with a suspicion of OSA.

## INTRODUCTION

Obstructive sleep apnea (OSA) is a relevant health problem because of its high prevalence in the general population and its significant morbidity and mortality^1^^-^5. Continuous positive airway pressure (CPAP) is a cost-effective treatment commonly prescribed in patients with OSA since it reduces daytime sleepiness, rates of motor vehicle accidents, and blood pressure^6^^-^9. Polysomnography in the sleep laboratory has been recommended for the diagnosis of OSA and CPAP titration^10^^,^^11^. Given this approach challenges the capacity of sleep laboratories and health system resources^12^, the need for low cost and less time-consuming diagnostic procedures becomes imperative. Since OSA treatment provides many benefits to patients, it is important to develop simpler strategies (than traditional PSG) for prompt diagnosis and access to treatment. In this regard, pulse oximetry has been extensively evaluated as an alternative diagnostic tool for OSA. Though, published guidelines are controversial as to whether oximetry can be used to diagnose and indicate CPAP treatment in patients with clinical suspicion of OSA^13^^,^^14^.

There is scarce information on whether a model based on clinical history and oximetry is a suitable approach to decide CPAP treatment in adult patients with suspicion of OSA^15^. We hypothesized that physicians experienced in the treatment of sleep-related breathing disorders could indicate a CPAP trial in patients with clinical suspicion of OSA and abnormal oximetry with low chance of error (by error we mean prescribing CPAP to patients who do not they required such treatment). Therefore, the aim of this large multicenter study was to assess the accuracy of CPAP treatment indication based on clinical and nocturnal oximetry data compared with PSG. The secondary objective was to evaluate predictors of CPAP indication among the observers who based their decision on pulse oximetry.

## METHODS

This was a retrospective multicenter observational study involving seven sleep laboratories in five large cities of Argentina: Ciudad Autónoma de Buenos Aires (Hospital Alemán, Hospital Británico, Hospital de Clínica), Córdoba (Centro Dr. Lázaro Langer), Mendoza (Instituto Médico Insares), Salta (Hospital Privado Santa Clara de Asis) and Paraná (Centro Privado de Medicina Respiratoria de Paraná). It was approved by the Ethics Committee of Hospital Alemán.

### Patient selection

**Figure 1:**
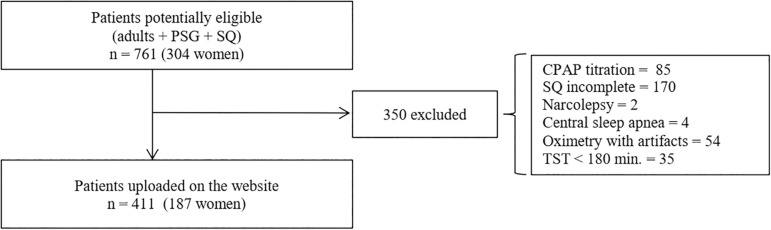
Flow chart for patient selection (PSG = Polysomnography; SQ = Sleep Questionnaires; TST = Total Sleep Time).

On a database of 761 patients (304 women) studied in the Hospital Aleman’s sleep laboratory between 2013 and 2015, an intentional sampling was carried out in order to have a similar proportion of men and women with a hypopnea apnea index (AHI) less than 5 and with mild, moderate and severe OSA. Patients over 18 years of age who had undergone PSG and filled out a standardized sleep questionnaire (SQ) were preselected. Exclusion criteria were: patients with a total sleep time of less than 180 minutes, PSG with CPAP titration, oximetry with artifacts by disconnections or finger probe displacement more than 5% of total recording time, subjects with suspected narcolepsy or central sleep apnea by PSG^16^ and patients who did not complete the questionnaire or chose two answers in some of the questions. 411 patients were included in the study. Figure 1 shows the flow chart for patient selection.

### Measurements

The patients selected for this study had a PSG with a computerized polysomnographic system (Akonic, Buenos Aires, Argentina) which included: electroencephalogram (F4/A1, C4/A1, O2/A1), electrooculogram, chin electromyogram, leg electromyogram, electrocardiogram, airflow by nasal pressure and an oral thermistor, thoracic and abdominal movements (piezoelectric sensors), snoring, SO2 and body position. PSG interpretation was performed according to published criteria^17^. OSA was defined as an apnea/hypopnea index (AHI) ≥ 5. Pulse oximetry from PSG was recorded using a finger probe (Nonin, OEM III) and the data storage rate was 1 Hz. Nail polish was removed to avoid interferences in SO_2_ readings. Software on PSG performed automatic SO_2_ analysis and excluded areas with artifacts (artifact time). Then, the program calculated the oxygen desaturation index (ODI) ≥ 3% (ODI3: mean number of O_2_ desaturations ≥ 3% per hour of valid analyzed recording) using the valid total recording time (total recording time - artifact time). ODI3 was selected to assess pulse oximetry as it has shown the best intraclass correlation coefficient with AHI and therefore could be used as surrogate for AHI^18^. Pulse oximetry suggestive of OSA was defined as the presence of a sawtooth pattern in one or more sectors of the oxygen saturation curve plus an oxygen desaturation index ≥ 3% (ODI3) > 5^19^.

Upon arrival to the sleep lab, the patients completed a standardized sleep questionnaire (SQ) that included the Spanish versions of the Epworth Sleepiness Scale (ESS)^20^, the Berlin questionnaire^21^^,^^22^, relevant medical history and medication.

Comorbidities such as hypertension, coronary disease, ischemic or hemorrhagic stroke or diabetes type II, were considered when patients reported them, or if they received any medication for their condition. Restless leg syndrome was assumed when patients complained of unpleasant sensations in the lower limbs before sleeping and reported behaviors to alleviate them. Tiredness was regarded as a symptom when it was described on a daily basis. Insomnia was defined both as difficulty falling asleep or waking up while sleeping and delay in returning to sleep more than 5 times a month.

### Study procedure

The database with all the information necessary for decision making was available on a website specifically designed for this research. Researchers could access it through username and password to see clinical data, questionnaires, oximetry or PSG data and treatment decision options.

Three to six months before starting the study, all researchers received written instructions to access and use the website tools so that they could train with twenty clinical cases to understand work dynamics. To help decide treatment with CPAP, all observers used a summary sleep questionnaire (SSQ). Eight physicians with at least 10 year experience in the management of patients with sleep-related breathing disorders independently logged onto a website and were blinded to any patient identification data or indication of CPAP. Seven observers (one per center) who could only access the clinical data (SSQ), curve and pulse oximetry results were collectively referred to as the test method (Os-SO_2_), while the eighth observer, who also had full access to all indicators of PSG, was the reference method (O-PSG). Once observers assessed the information available on the website, they had to choose between three CPAP treatment options (“yes”, “no” or “do not know”). The term “do not know” could be selected when the observers had doubts in the prescription of CPAP or could not make the decision with the information available. Observers 1 to 7 could indicate CPAP treatment if the patients met clinical criteria and their oximetry was compatible with OSA. O-PSG and Os-SO2 supported CPAP treatment decision on their experience and current published guidelines^23^^,^^24^.

### Statistical analysis

According to the distribution of the variables, the mean and standard deviation or the median and the percentiles 25-75% were reported. Differences between proportions were calculated by Chi Square test.

The accuracy of the Os-SO_2_ (test method) for the indication of CPAP was evaluated by calculating the sensitivity (S), specificity (Sp), positive and negative likelihood ratio (PLR, NLR) in each observer for both the total group and the more or less symptomatic patients. The more symptomatic patients were defined as the presence of Epworth greater than 10 and / or at least one comorbidity, while the less symptomatic subjects were those with an Epworth less than 11 and without comorbidities^25^.

Multiple logistic regression analysis was used to assess the association between CPAP indication most frequently made by Os-SO2 in each patient (depending variable: CPAP: 1 = Yes, 0 = No/Do not know) and age, gender (1 = Male, 0 = Female), BMI, sleepiness (1 = ESS > 10, 0 = ESS < 11), comorbidities (1 = Yes, 0 = No), insomnia (1 = Yes, 0 = No), restless leg syndrome (1 = Yes, 0 = No) and ODI3 ≥ 15 (1 = ODI3 ≥ 15, 0 = ODI3 < 15) (independent variables). ODI3 ≥ 15 was chosen because this cut-off point has shown good performance to diagnose moderate to severe OSA^19^. The statistical analysis was performed with the following software programs: MedCalc Software, Version 18, Mariakerke, Belgium.

## RESULTS

Of 761 potentially eligible patients, 350 were excluded for different reasons (see Figure 1). Thus, there were 411 patients for the final analysis (Figure 1). The characteristics of the study population are shown in Table 1. The median age was 54 years, 55.5% (228) were men, 76.4% (314) had OSA (AHI ≥ 5), and 31.4% (129) were obese. As for OSA severity categories, similar percentages were found: no OSA (23.6%), mild OSA (23.8%), moderate OSA (25.6%) and severe OSA (27%) (*p* 0.8). ESS > 10 was present in 38.2% of patients and tiredness was reported by 54%.

**Table 1:** Characteristics of the study population.

Patient number	411
Male	224 (54.5)
Female	187 (45.5)
Age (years)	54 (42 - 64)
Body mass index (BMI kg./m^2^)	27.8 (24.7 - 31.2)
Prevalence of OSA (AHI ≥ 5)	314 (76.4)
Epworth	9 (6 - 13)
Epworth > 10	157 (38.2)
Tiredness	222 (54)
Comorbidities	
- Hypertension	135 (32.9)
- Coronary heart disease	22 (5.35)
- Ischemic or hemorrhagic stroke	9 (2.2)
- Diabetes type II	101 (24.6)
Restless leg syndrome	107 (24.6)
Insomnia	114 (27.8)
Polysomnography	
- TRT (total recording time, min)	395.5 (383.5 - 420)
- TST (total sleep time, min.)	334.4 (298.7 - 362.7)
- SE (sleep efficiency)	85 (79 - 90)
- TNREM (min.)	277.3 (249.9 - 302.4)
- TREM (min.)	53 (37.3 - 69.3)
- AHI (Apnea/Hypopnea Index)	16.5 (6.2 - 32)
- T90 (%)	2.2 (0.2 - 12.1)
- SO2 median (%)	92.8 (91.5 - 94.2)
- ODI3	9.6 (3.8 - 20.8)
Severity of OSA (%)	
- AHI ≥ 5 - < 15	98 (23.8)
- AHI ≥ 15 - < 30	105 (25.6)
- AHI ≥ 30	111 (27)

Data are presented as median (25-75 % percentiles), or n (%). TNREM: total stages N1+N2+N3; TREM: total amount of REM sleep. ODI3: oxygen desaturation index ≥ 3%. T90 (%): percentage of TRT at SO2 < 90%.

Table 2 shows the patients´ characteristics according to symptoms. The more symptomatic patients (ESS > 10 or one comorbidity) were older and presented a higher prevalence of obesity, tiredness, insomnia and RLS compared to the less symptomatic subjects. On the other hand, OSA prevalence, its severity, and oximetry parameters were similar in both groups.

**Table 2:** Comparison of more or less symptomatic patients.

	More symptomatic	Less symptomatic	p
Patient number (%)	276 (67.1)	135 (32.8)	< 0.001
Male %	48.2	67.4	0.0002
Age (years)	57 (46 - 66)	48 (38 - 58)	< 0.001
Body mass index (BMI kg./m^2^)	28.1 (24.7 - 31.6)	27.6 ( 24.7 - 29.8)	0.10
Obesity %	35.6	8.9	< 0.001
Prevalence of OSA % (AHI ≥ 5)	74.6	80	0.23
Epworth	11 (6 - 15)	7 (4 - 8)	< 0.001
Tiredness %	60.1	41.5	< 0.001
Restless leg syndrome %	34.8	4	< 0.001
Insomnia %	30.8	21.5	0.048
Polysomnography			
- TRT (total recording time, min)	397 (384 - 420)	395 (381 - 416)	0.3
- TST (total sleep time, min.)	334 (302 - 363)	334.5 (295 - 361)	0.76
- SE (sleep efficiency)	0.85 (0.78 - 0.9)	0.85 (0.8 - 0.89)	0.63
- TNREM (min.)	277.3 (251 - 304)	277 (246 - 298)	0.48
- TREM (min.)	52.4 (37.4 - 69.3)	53.3 (37.3 - 69.4)	0.78
- AHI (Apnea/Hypopnea Index)	17.4 (4.6 - 33.3)	14.5 (7.2 - 28.2)	0.42
- T90 (%)	2.6 (0.2 - 15.6)	1.6 (0.1 - 7.7)	0.15
- SO2 median (%)	92.8 (91.3 - 94)	92.8 (91.9 - 94.4)	0.08
- ODI3	9.6 (3.8 - 23.7)	9.4 (3.3 - 19.6)	0.38
Severity of OSA			
- AHI ≥ 5 - < 15 %	20.6	30.4	0.03
- AHI ≥ 15 - < 30 %	25	26.7	0.71
- AHI ≥ 30 %	29	23	0.2

Data are presented as median (25-75 % percentiles), or n (%). TNREM: total stages N1+N2+N3; TREM: total amount of REM sleep. ODI3: oxygen desaturation index ≥ 3%; T90 (%): percentage of TRT at SO2 < 90%.

O-PSG indicated CPAP to 225 subjects (54.7%) of which 85% presented moderate to severe OSA (AHI median 29, interquartile range 19 - 43) and 80% had either sleepiness or at least one comorbidity.

S, Sp and PLR / NLR for Os-SO_2_ are shown in Table 3. In the whole group (WG), Os-SO_2_ had average S, Sp and PLR of 61.7%, 93.4% and 19.3, respectively. Os-SO_2_ had lower S, greater Sp and PLR for CPAP indication in more symptomatic patients than those with fewer symptoms (mean S: 60 *versus* 68%, *p* 0.046; mean Sp: 98.4 *versus* 88%, *p*<0.01; mean PLR: 38.9 *versus* PLR 10, *p*<0.01).

**Table 3:** AUC-ROC, sensitivity, specificity and positive/negative likelihood ratios from Os-SO_2_

**Whole group (n = 411)**								
**Os-SO_2_**	**Sensitivity**		**Specificity**		**PLR**		**NLR**	
	**Range**	**Mean (CI 95%)**	**Range**	**Mean (CI 95%)**	**Range**	**Mean (CI 95%)**	**Range**	**Mean (CI 95%)**
O1 - O7	45.3 - 78.7	61.7 (49.3 - 74.1)	81.7 - 98.4	93.4 (87.1 - 99.7)	5.3 - 34.7	18.3 (7.7 - 29)	0.26 - 0.56	0.4 (0.29 - 0.52)
**More symptomatic patients (Epworth > 10 or at least one comorbidity) (n=276)**								
**Specificity**	**Sensitivity**		**Specificity**		**PLR**		**NLR**	
	**Range**	**Mean (CI95%)**	**Range**	**Mean (CI95%)**	**Range**	**Mean (CI95%)**	**Range**	**Mean (CI95%)**
O1 - O7	44.7 - 75.4	60 (48 - 72)	93.8 - 100	98.4 (96.2 - 100)	12.2 - 71.5	38.9 (17.6 - 60)	0.26 - 0.55	0.4 (0.29 - 0.52)
**Less symptomatic patients (Epworth < 11 without comorbidities) (n=135)**								
**Os-SO_2_**	**Sensitivity**		**Specificity**		**PLR**		**NLR**	
	**Range**	**Mean (CI95%)**	**Range**	**Mean (CI95%)**	**Range**	**Mean (CI95%)**	**Range**	**Mean (CI95%)**
O1 - O7	44.7 - 87	68 (53 - 83)	68.5 - 96.6	88 (77 - 99)	2.9 - 14.2	10 (4.8 - 15.4)	0.18 - 0.54	0.35 (0.2 - 0.48)

Os-SO_2_: observers who use the clinical history and oximetry to indicate CPAP (O1 - 7); CI95%: confidence interval 95%; PLR/NLR: positive and negative likelihood ratio.

Two Os-SO_2_, reported the highest number of false positive (FP) and negative (FN) cases. Table 4 compares FP and FN with true negative (TN) and positive (TP) cases. FP patients showed an AHI, an ODI3, and a BMI higher than TN cases. On the other hand, FN cases had an AHI, an ODI3 and a lower BMI than TP subjects. TP and TN subjects had a clearly abnormal oximetry (sawtooth pattern and mean ODI3 34) or one without cyclical variations in the SO2 curve (mean ODI3 1.4).

**Table 4:** Characteristics of false positive and negative patients of whole group (O4 and O5)

	**Observer 4**		
**Variables**	**False positive (n=34)**	**True negative (n=152)**	**p**
AHI	16.3 (11.3 - 21.1)	3 (1.1 - 7.8)	< 0.001
-AHI < 5	3 (8.8)	-	-
-AHI ≥ 5 - < 15	11 (32.4)	-	-
-AHI ≥ 15	20 (58.8)	-	-
BMI (Kg/m^2^)	27.1 (25.7 - 29.7)	25.2 (22.5 - 28.1)	0.0073
Tiredness	13 (38.2)	73 (48)	0.3
Epworth > 10	5 (14.7)	48 (31.6)	0.049
Comorbidities	1 (2.9)	57 (37.5)	< 0.001
ODI3	10 (7.3 - 15.5)	1.4 (1.4 - 4.7)	< 0.001
	**Observer 5**		
**Variables**	**False negative (n=123)**	**True positive (n=102)**	**p**
AHI	21.2 (14.1 - 29)	45.5 (32.7 - 57.1)	< 0.001
-AHI < 5	0 (0)	-	-
-AHI ≥ 5 - < 15	32 (26)	-	-
-AHI ≥ 15 - <30	64 (52)	-	-
-AHI ≥ 30	27 (22)	-	-
Tiredness	80 (65)	56 (54.9)	0.12
BMI (Kg/m^2^)	28.4 (25.7 - 31.6)	31.2 (28.4 - 35.1)	< 0.001
Epworth > 10	51 (41.5)	53 (52)	0.12
Comorbidities	76 (61.8)	62 (60.8)	0.88
ODI3	12.2 (7.9 - 16.9)	34.4 (25.5 - 48)	< 0.001

Data are presented as median (25-75% percentiles) or percentage (%). AHI: Apnea/Hypopnea Index; ODI3: oxygen desaturation index ≥ 3%.

Table 5 shows the predictors of CPAP indication. An ODI3 ≥ 15 was the most powerful predictor used for therapeutic decision making (OR: 145, 95%CI 57 - 366, *p*<0.0001). Also, being male (OR: 2.56, 95% CI 1.1 - 5.9, *p* 0.026, having a higher BMI (OR: 1.14, 95% CI 1.03 - 1.25, *p* 0.0064), an ESS > 10 (OR: 2.5, 95%CI 1.04 - 6.1, *p* 0.04) and some comorbidities (OR: 3.2, 95%CI 1.35 - 7.6, *p* 0.0082) were independently associated with CPAP indication.

**Table 5:** Predictors of CPAP indication from Os-SO_2_.

Parameters	Coefficient	Std. Error	Odds ratio	p
Dependent variable: CPAP indication				
Independent variables				
-Age	0.013	0.016	1.01 (0.98 - 1.04)	0.40
-BMI	0.13	0.047	1.14 (1.04 - 1.25)	0.0064
-Gender	0.94	0.42	2.5 (1.12 - 5.9)	0.0265
-Epworth > 10	0.93	0.45	2.5 (1.04 - 6.1)	0.04
-Tiredness	0.55	0.46	1.73 (0.7 - 4.2)	0.23
-Comorbidities	1.16	0.44	3.2 (1.35 - 7.6)	0.0082
-Insomnia	0.025	0.49	1.03 (0.39 - 2.7)	0.96
-Restless leg Syndrome (RLS)	0.43	0.53	1.5 (0.54 - 4.3)	0.42
-ODI3 ≥ 15	5	0.47	145 (57 - 366)	< 0.0001

Os-SO_2_: observers who use the clinical history and oximetry to indicate CPAP.

We assess the direct costs of an initial approach based on oximetry or polysomnography, knowledge (guidelines) and experience of physicians to indicate CPAP treatment in more symptomatic patients. For this, we considered the decision of treatment with CPAP most frequently made by Os-SO_2_, which had 80 false-negative subjects and no false positive case, and the average costs in the local market. Based on these data, we could have saved US$ 24200 (Table 6).

**Table 6:** Cost-benefit analysis of an initial diagnostic approach with polysomnography or oximetry.

Initial diagnostic strategy	n	Unit price ^a^	Final cost ^a^	
**1) Polysomnography**				
First visit	411	15	6165	
Polysomnography	411	200	82200	
Second visit	411	15	6165	
CPAP trial (auto-CPAP)	225	150	33750	
**Total cost**			**128280**	
**2) Nocturnal oximetry**				
First visit	411	15	6165	
Nocturnal oximetry (more symptomatic patients)	276	50	13800	
Polysomnography (less symptomatic patients)	135	200	27000	
Second visit	411	15	6165	
CPAP trial (auto-CPAP) (more symptomatic)	99	150	14850	
CPAP trial (auto-CPAP) (less symptomatic)	46	150	6900	
Polysomnography (more symptomatic without CPAP				
indication by oximetry)	80	200	16000	
Third visit	80	15	1200	
CPAP trial (auto-CPAP) in false negative cases by				
oximetry	80	150	12000	
**Total cost**			**104080**	
**Cost savings**				**24200**

n: number of studies. a: expressed as US $ dollars

## DISCUSSION

The main finding of this multicenter study was that a strategy based on clinical history and pulse oximetry allowed sleep specialists to reliably indicate CPAP treatment for approximately 60% of the more symptomatic patients who required such therapy according to clinical criteria and PSG. This approach showed varying sensitivity in Os-SO_2_, both in the total group (45 - 79%) and subgroups of more (45 - 75%) or less symptomatic (45 - 87%) patients. This interobserver heterogeneity possibly reflects the complexity of the CPAP treatment decision-making procedure, which involves a wide range of variables, such as symptoms (sleepiness or tiredness being the most important), associated comorbidities and physician´s experience with OSA patients and the interpretation of pulse oximetry. In our patient population, the pretest probability, (i.e. CPAP recommendation by PSG) was 55% in the total group, 65% in the more symptomatic patients and 34% in those with fewer symptoms. Depending on the PLR, the posttest probability of obtaining a true positive diagnosis ranged from 60 to 90% in oligosymptomatic subjects, 80 to 97% in the total group and 95 to 99% in the more symptomatic patients^26^. Although the CPAP indication rate varied among Os-SO_2_ observers, Sp was similar (94 to 100%, *p* 0.7) and PLR was greater than 10 in more symptomatic patients. The low proportion of false positive subjects makes this approach a consistent tool. In our cohort, observer 4 showed the highest number of FP cases. However, when analyzing these 34 cases, 30 patients could have had CPAP indication according to the published guidelines^24^, since 20 had moderate OSA and 10 were mild forms of OSA with associated symptoms. Thus, there were only 4 cases who did not really require CPAP. Eventually, a CPAP trial could have only caused minor side effects without serious risk to the patient. Also, during the CPAP trial, treatment adherence and response are tested, and if necessary, the treatment mode could be changed. The drawback with an initial diagnostic approach based on nocturnal oximetry in the entire patient sample was that there were an average of 40% of false negative cases (i.e., O-PSG indicated CPAP but Os-SO_2_ did not). In this way, patients suspected of obstructive sleep apnea but with inconclusive oximetry would require a second diagnostic sleep study with the consequent potential increase in costs and treatment delay. Despite these disadvantages, if this strategy had been adopted only in more symptomatic patients, the health costs could have been reduced by almost 20% compared to the traditional polysomnography-based approach (see Table 6).

We recently published^15^ a study where two blind independent physicians agreed on CPAP therapy according to PSG (gold standard) or oximetry (alternative method) results and symptoms. The S was higher (80 - 92%) and the Sp similar (92 - 96%) to the current study. The differences between both studies could be related to population differences (moderate to severe OSA: 70% *vs.* 52%), the number of observers who participated in both studies (seven *versus* one) and the fact that observers´ decisions were based exclusively on CPAP treatment guidelines without resorting to their experience.

Our results are in line with previous clinical validation studies of oximetry. Mulgrew et al.^27^ showed that in patients with a Sleep Apnea Clinical Score ≥ 15, Epworth ≥ 10 and ODI4 ≥ 15, an ambulatory approach based on auto-CPAP plus oximetry was similar to PSG in terms of CPAP titration and clinical outcomes. Antic et al.^28^ observed that in patients with frequent snoring plus ESS ≥ 8 and ODI3 > to 27, a nurse model of care based on oximetry and auto-CPAP showed similar results to physician care based on PSG in terms of clinical improvement and adherence to CPAP treatment. Similarly, Chai-Coetzer et al.^29^ demonstrated treatment under a primary care model compared with a specialist model did not result in worse sleepiness scores, quality of life or CPAP compliance in patients with high pretest for moderate to severe OSA (clinical score ≥ 5, ODI3 ≥ 16 and ESS > 7 or persistent hypertension with 2 or more drugs). These findings are consistent with NICE guidelines which state that moderate to severe OSA can be diagnosed through clinical history and pulse oximetry performed at the patient’s home^30^.

This study has several strengths. It is a multicenter study of a large sample of patients and a similar number of men and women. Also, the proportion of subjects without or with mild, moderate or severe OSA was similar. All patients had a standardized sleep questionnaire and a PSG. Finally, the observers who participated were blind and the therapeutic decision to or not to treat with CPAP was based on each observer´s knowledge and experience so as to simulate a real-life scenario. However, this clinical research presents some limitations. First, its retrospective design and selection bias limits its generalization. Second, this simple strategy requires training in the management of patients with sleep apnea and analysis of oximetry, therefore it cannot be extrapolated to general practitioners or primary care physicians.

In conclusion, a tool based on clinical data and pulse oximetry allowed to indicate CPAP treatment reliably in most symptomatic patients with suspicion of OSA. This approach would be useful in the initial management of OSA when access to specialized sleep medicine centers is limited for different reasons. Future studies are necessary to confirm these results in a real life scenario.

## References

[1] Franklin KA, Lindberg E (2015). Obstructive sleep apnea is a common disorder in the population-a review on the epidemiology of sleep apnea. J Thorac Dis.

[2] Peppard PE, Young T, Palta M, Skatrud J (2000). Prospective study of the association between sleep-disordered breathing and hypertension. N Engl J Med.

[3] Newman AB, Nieto J, Guirdry U, Lind BK, Redline S, Pickering TG, Sleep Heart Health Study Research Group (2001). Relation of sleep-disordered breathing to cardiovascular disease risk factors: the Sleep Heart Health Study. Am J Epidemiol.

[4] Lavie P, Herer P, Peled R, Berger I, Yoffe N, Zomer J (1995). Mortality in sleep apnoea patients: multivariate analysis of risk factors. Sleep.

[5] Tufik S, Santos-Silva R, Taddei JA, Bittencourt LR (2010). Obstructive sleep apnea syndrome in the Sao Paulo Epidemiologic Sleep Study. Sleep Med.

[6] Ayas NT, Marra C (2005). Continuous positive airway pressure therapy for obstructive sleep apnea syndrome: do the dollars make sense?. Sleep.

[7] Engleman HM, Martin SE, Kingshott RN, Mackay TW, Deary IJ, Douglas NJ (1998). Randomised placebo controlled trial of daytime function after continuous positive airway pressure (CPAP) therapy for the sleep apnoea/hypopnoea syndrome. Thorax.

[8] Cassel W, Ploch T, Becker C, Dugnus D, Peter JH, von Wichert P (1996). Risk of traffics accidents in patients with sleep-disordered breathing: reduction with nasal CPAP. Eur Respir J.

[9] Faccenda JF, Mackay TW, Boon NA, Douglas NJ (2001). Randomized placebo-controlled trial of continuous positive airway pressure on blood pressure in the sleep apnea-hypopnea syndrome. Am J Respir Crit Care Med.

[10] (1994). Indications and standards for use of nasal continuous positive airway pressure (CPAP) in sleep apnea syndromes. American Thoracic Society. Official statement adopted March 1944. Am J Respir Crit Care Med.

[11] (1997). Practice parameters for the indications for polysomnography and related procedures. Polysomnography Task Force, American Sleep Disorders Association Standards of Practice Committee. Sleep.

[12] Flemons WW, Douglas NJ, Kuna ST, Rodenstein DO, Wheatley J (2004). Access to diagnosis and treatment of patients with suspected sleep apnea. Am J Respir Crit Care Med.

[13] Corral-Peñafiel J, Pepin JL, Barbe F (2013). Ambulatory monitoring in the diagnosis and management of obstructive sleep apnoea syndrome. Eur Respir Rev.

[14] Fleetham J, Ayas N, Bradley D, Fitzpatrick M, Oliver TK, Morrison D (2011). Canadian Thoracic Society Sleep Disordered Breathing Committee. Canadian Thoracic Society 2011 guideline update: diagnosis and treatment of sleep disordered breathing. Can Respir.

[15] Nigro CA, Dibur E, Rhodius E (2012). Accuracy of the clinical parameters and oximetry to initiate CPAP in patients with suspected obstructive sleep apnea. Sleep Breath.

[16] White DP, Kryger MH, Roth T, Dement WC, eds (2005). Central sleep apnea. Principles and practice of sleep medicine.

[17] Berry RB, Budhiraja R, Gottlieb DJ, Gozal D, Iber C, Kapur VK, American Academy of Sleep Medicine (2012). Rules for scoring respiratory events in sleep: update of the 2007 AASM Manual for the Scoring of Sleep and Associated Events. Deliberations of the Sleep Apnea Definitions Task Force of the American Academy of Sleep Medicine. J Clin Sleep Med.

[18] Nigro CA, Dibur E, Rhodius E (2011). Pulse oximetry for the detection of obstructive sleep apnea syndrome: can the memory capacity of oxygen saturation influence their diagnostic accuracy?. Sleep Disord.

[19] Oeverland B, Skatvedt O, Kvaerner KJ, Akre H (2002). Pulseoximetry: sufficient to diagnose severe sleep apnea. Sleep Med.

[20] Chica-Urzola HL, Escobar-Córdoba F, Eslava-Schmalbach J (2007). Validating the Epworth sleepiness scale. Rev Salud Publica (Bogota).

[21] Netzer NC, Stoohs RA, Netzer CM, Clark K, Strohl KP (1999). Using the Berlin questionnaire to identify patients at risk for the sleep apnea syndrome. Ann Intern Med.

[22] Varela B, Nigro CA, Dibur E, Malnis S, Rhodius EE (2010). Utilidad del cuestionario de Berlín y el Mallanpati para el diagnóstico del síndrome apnea/hipopnea obstructiva del sueño. Revista Americana de Medicina Respiratoria.

[23] Lloberes P, Durán-Cantolla J, Martínez-García MÁ, Marín JM, Ferrer A, Corral J (2011). Diagnosis and treatment of sleep apnea-hypopnea syndrome. Spanish Society of Pulmonology and Thoracic Surgery. Arch Bronconeumol.

[24] Nogueira F, Nigro C, Cambursano H, Borsini E, Silio J, Avila J (2013). Practical guidelines for the diagnosis and treatment of obstructive sleep apnea syndrome. Medicina (B Aires).

[25] Masa JF, Duran-Cantolla J, Capote F, Cabello M, Abad J, Garcia-Rio F, Spanish Sleep Network (2015). Efficacy of home single-channel nasal pressure for recommending continuous positive airway pressure treatment in sleep apnea. Sleep.

[26] Fagan TJ (1975). Letter: Nomogram for Bayes theorem. N Engl J Med.

[27] Mulgrew AT, Fox N, Ayas NT, Ryan CF (2007). Diagnosis and initial management of obstructive sleep apnea without polysomnography: a randomized validation study. Ann Intern Med.

[28] Antic NA, Buchan C, Esterman A, Hensley M, Naughton MT, Rowland S (2009). A randomized controlled trial of nurse-led care for symptomatic moderate-severe obstructive sleep apnea. Am J Respir Crit Care Med.

[29] Chai-Coetzer CL, Antic NA, Rowland LS, Reed RL, Esterman A, Catcheside PG (2013). Primary care vs specialist sleep center management of obstructive sleep apnea and daytime sleepiness and quality of life: a randomized trial. JAMA.

[30] National Institute for Health and Care Excellence (NICE) (2008). Continuous positive airway pressure for the treatment of obstructive sleep apnoea/hypopnoea syndrome.Technology appraisal guidance.

